# Chiral *versus* achiral crystal structures of 4-benzyl-1*H*-pyrazole and its 3,5-di­amino derivative

**DOI:** 10.1107/S2056989024006182

**Published:** 2024-06-28

**Authors:** Emily R. Hayward, Matthias Zeller, Gellert Mezei

**Affiliations:** a Western Michigan University, Department of Chemistry 1903 W Michigan Ave Kalamazoo MI 49008 USA; b Department of Chemistry, Purdue University, 560 Oval Dr., West Lafayette, IN 47907, USA; Katholieke Universiteit Leuven, Belgium

**Keywords:** pyrazole, crystal structure, hydrogen-bonding motifs, chirality

## Abstract

The crystal structures of 4-benzyl-1*H*-pyrazole (C_10_H_10_N_2_, **1**) and 3,5-di­amino-4-benzyl-1*H*-pyrazole (C_10_H_12_N_4_, **2**) were measured at 150 K. Although its different conformers and atropenanti­omers easily inter­convert in solution by annular tautomerism and/or rotation of the benzyl substituent around the C(pyrazole)—C(CH_2_) single bond (as revealed by ^1^H NMR spectroscopy), **1** crystallizes in the non-centrosymmetric space group *P*2_1_. Within its crystal structure, the pyrazole and phenyl aromatic moieties are organized into alternating bilayers. Both pyrazole and phenyl layers consist of aromatic rings stacked into columns in two orthogonal directions. Within the pyrazole layer, the pyrazole rings form parallel catemers by N—H⋯N hydrogen bonding. Compound **2** adopts a similar bilayer structure, albeit in the centrosymmetric space group *P*2_1_/*c*, with pyrazole N—H protons as donors in N—H⋯π hydrogen bonds with neighboring pyrazole rings, and NH_2_ protons as donors in N—H⋯N hydrogen bonds with adjacent pyrazoles and other NH_2_ moieties. The crystal structures and supra­molecular features of **1** and **2** are contrasted with the two known structures of their analogs, 3,5-dimethyl-4-benzyl-1*H*-pyrazole and 3,5-diphenyl-4-benzyl-1*H*-pyrazole.

## Abstract 2

Despite the facile inter­conversion of its conformers in solution, 4-benzyl-1*H*-pyrazole adopts a chiral crystal structure (space group *P*2_1_). Its 3,5-di­amino derivative, however, crystallizes in the centrosymmetric space group *P*2_1_/*c*. In both crystal structures, the aromatic moieties are organized in alternating bilayers in which they stack in parallel columns in two orthogonal directions, and the pyrazole units form catemer motifs by N—H⋯N and N—H⋯π hydrogen bonding, respectively.

## 1. Chemical context

1*H*-Pyrazole (pzH) is a chemically and thermally robust organic mol­ecule (Katritzky *et al.*, 2010 ▸). Hence, its function­alized derivatives have found widespread applications as pharmaceuticals, pesticides and dyes (Ahmed *et al.*, 2016 ▸ and references therein). Owing to its adjacent pair of N atoms, pyrazole derivatives are also very popular in coordination chemistry, especially for the construction of discrete polynuclear complexes (Al Isawi *et al.*, 2021 ▸ and references therein). Within the crystal packing of pyrazole derivatives with different substituents, N—H⋯N hydrogen bonding between pz moieties leads to either discrete hydrogen-bonded motifs, such as dimers, trimers, tetra­mers and hexa­mers, or polymeric catemers depending on the substituents (Alkorta *et al.*, 2006 ▸; Bertolasi *et al.*, 1999 ▸; Cammers & Parkin, 2004 ▸; Claramunt *et al.*, 2006 ▸; Foces-Foces *et al.*, 2000 ▸). In general, the overall crystal structure is the result of the inter­play of optimal shape packing and multiple different inter­molecular forces, including electrostatic inter­actions, hydro­phobic effects, aromatic inter­actions, hydrogen bonding with potential hydrogen-bond donor/acceptor substituents, halogen bonding and other non-covalent inter­actions.
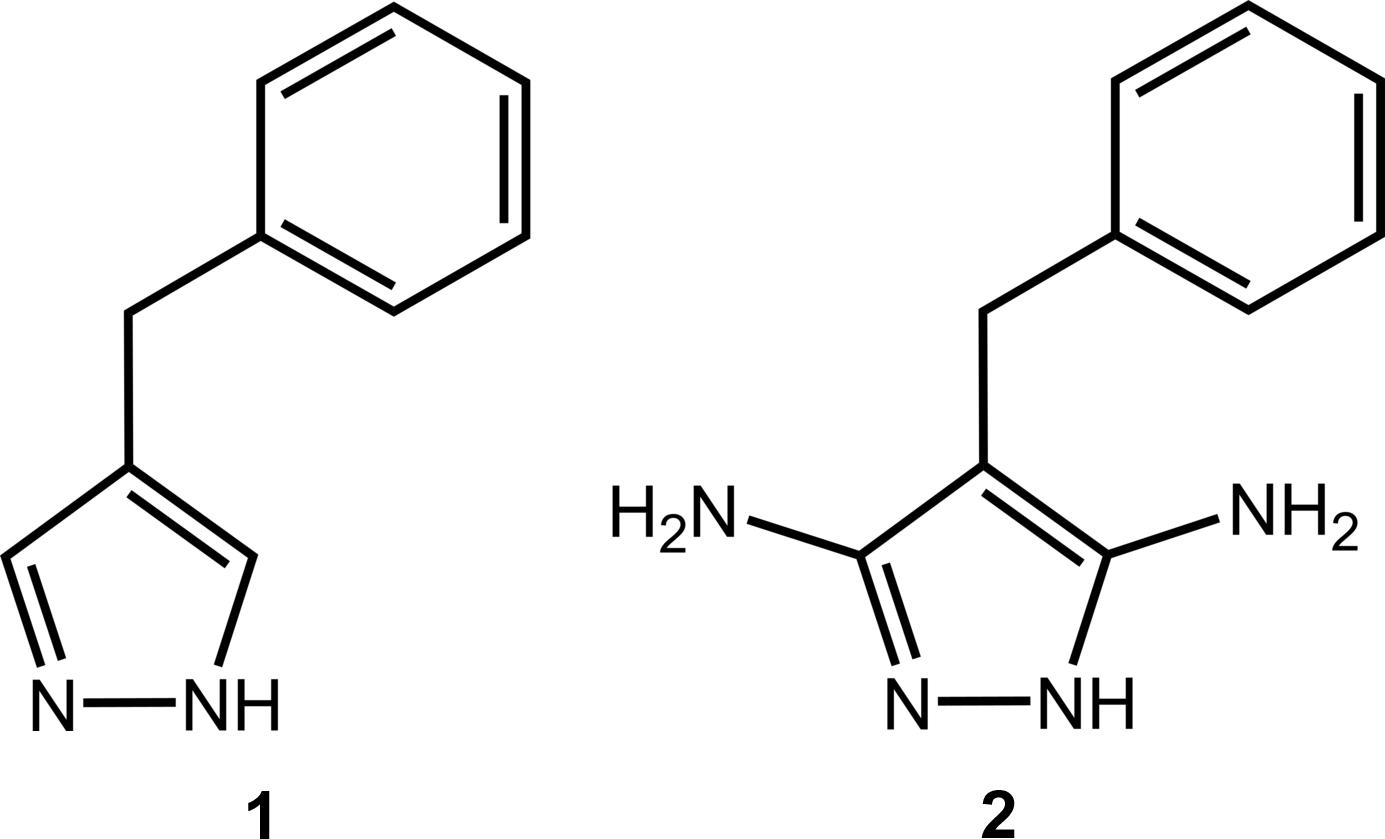


## 2. Structural commentary

**Figure 1:**
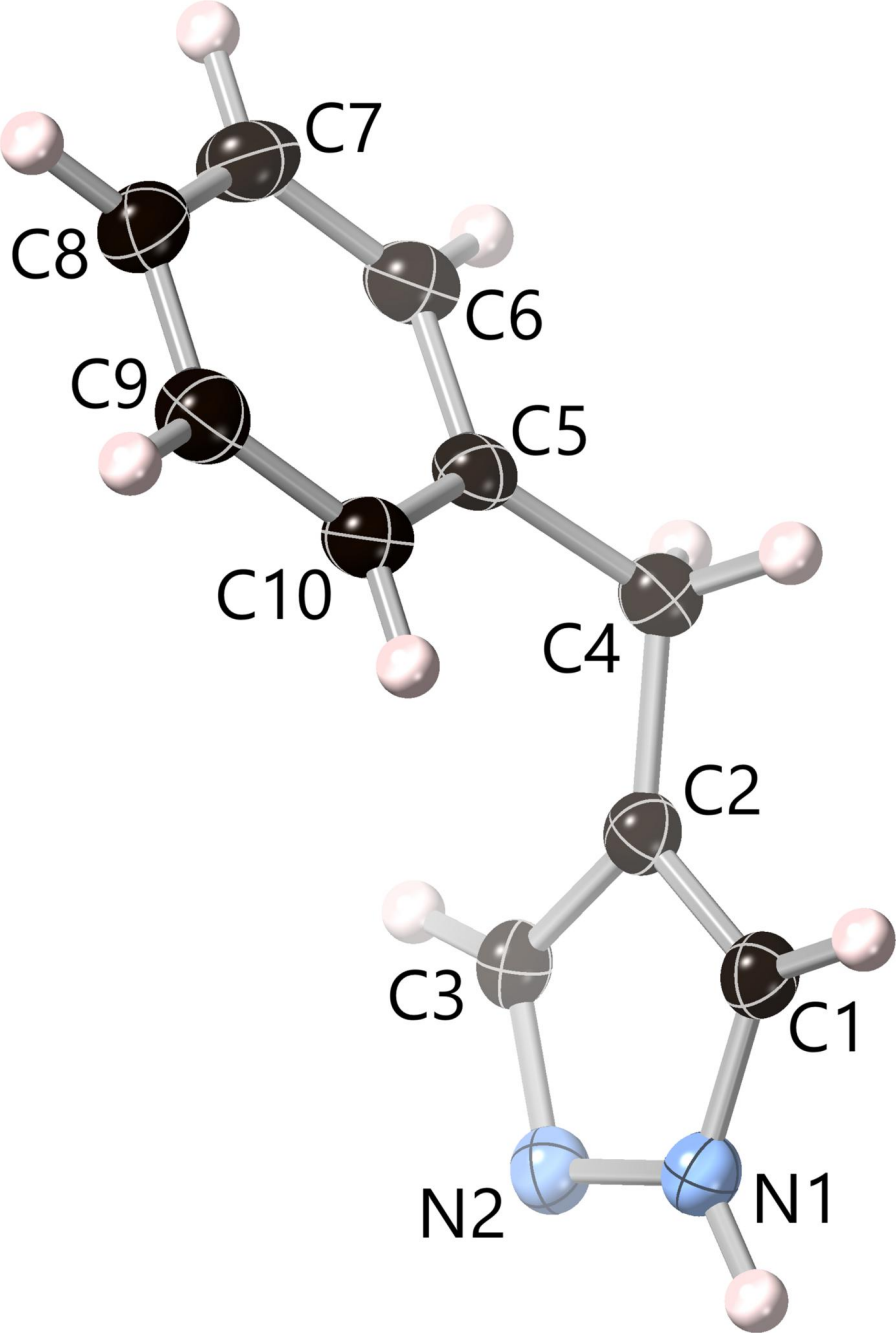
Displacement ellipsoid plot (50% probability) of the crystal structure of 4-benzyl-1*H*-pyrazole (**1**), showing the contents of the asymmetric unit.

Displacement ellipsoid plots of the crystal structures of **1** and **2** are shown in Figs. 1 ▸ and 2 ▸, respectively. Similarly to the parent pyrazole (La Cour & Rasmussen, 1973 ▸; Sikora & Katrusiak, 2013 ▸) and 4-fluoro­pyrazole (Ahmed *et al.*, 2023 ▸), the NH and N centers of the pz rings in **1** and **2** are not disordered and two distinct sets of C—N and C—C bond distances are observed. Thus, the C—N bond adjacent to N is shorter than the one adjacent to NH, whereas the C—C bond adjacent to N is longer than the one adjacent to NH (see supporting information). This is in contrast with other pyrazole derivatives, such as 4-phenyl­pyrazole (Reger *et al.*, 2003 ▸) and 4-halo­pyrazoles (halogen = Cl, Br, I; Rue & Raptis, 2021 ▸; Foces-Foces *et al.*, 1999 ▸; Rue *et al.*, 2023 ▸), where the N—H hydrogen atom is disordered over the two N atoms of the pyrazole unit. Otherwise, the N—N, C—N and C—C bond lengths in **1** and **2** are similar to those observed in related pyrazole derivatives. C—C—C bond angles between the pz, CH_2_ and Ph units are 63.85 (15)° in **1** and 65.65 (9)° in **2**, with pz/Ph centroid–centroid distances of 4.8294 (10) and 4.7376 (9) Å, respectively. While the dihedral angles between the pz and Ph units in **1** and **2** are not very disparate [86.00 (7) and 65.27 (4)°], the corresponding individual fold and twist angles are rather different. Specifically, the fold angle is much smaller in **1** [17.52 (12)°] than in **2** [76.12 (8)°], whereas the twist angle is much larger in **1** [65.00 (4)°] than in **2** [7.42 (6)°].

**Figure 3:**
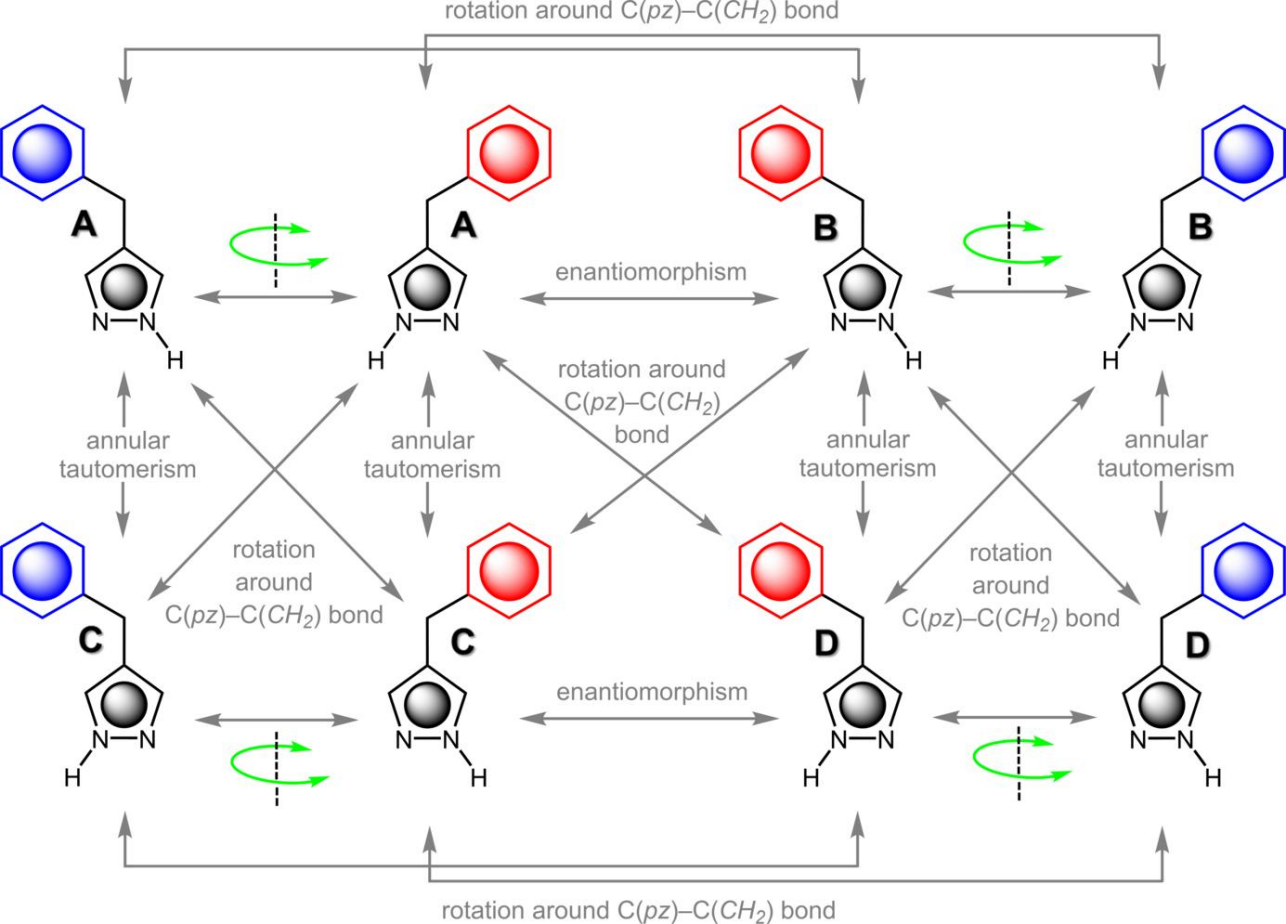
Inter­conversion between the different conformers, tautomers and atropenanti­omers of 4-benzyl-1*H*-pyrazole (**A**–**D**) by annular tautomerism and/or rotation of the benzyl moiety (red: *above* pz plane; blue: *below* pz plane) around the C—C bond between the pz and CH_2_ units.

**Figure 4:**
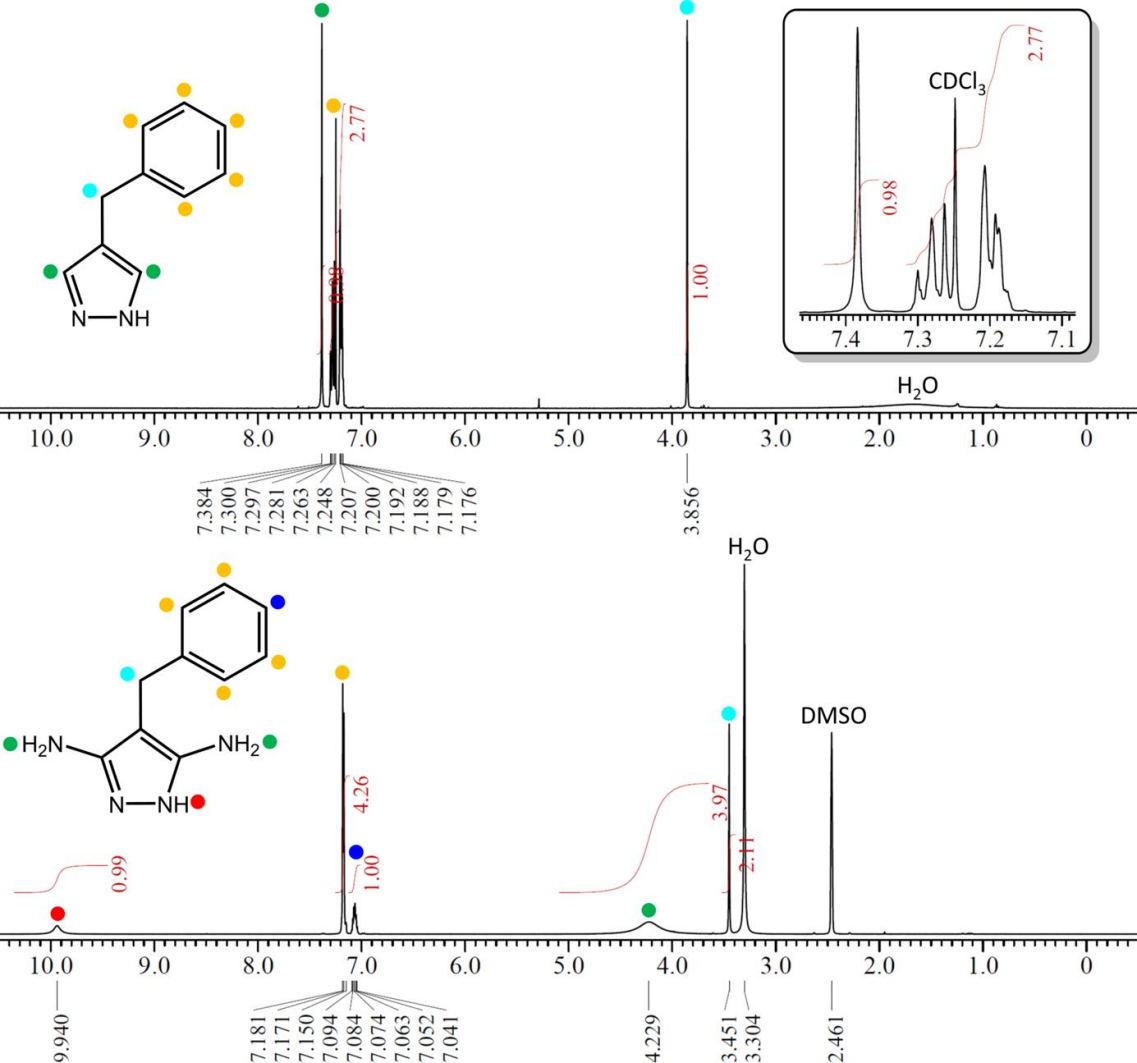
^1^H NMR spectra of **1** in CDCl_3_ (upper) and **2** in DMSO-*d*_6_ (lower) at ambient temperature. The signal for the NH proton of **1** is not detectable due to exchange with the solvent deuterium.

An inter­esting difference between the structures of **1** (*P*2_1_) and **2** (*P*2_1_/*c*) is related to their crystal symmetry. Although 4-benzyl-1*H*-pyrazole displays axial chirality (atropisomerism; Basilaia *et al.*, 2022 ▸) in the crystal structure described here, the two atropenanti­omers can inter­convert in solution by rotation of the benzyl substituent around the C(pz)—C(CH_2_) single bond (Fig. 3 ▸). Even if the direct conversion of conformer **A** to conformer **B** by rotation of the benzyl group from one side to the other side of the pz moiety would meet a significant barrier (caused by bulky substituents on the pz ring), **A** can still convert to **B** through its annular tautomer **C**. The latter converts to **B** by a same-side rotation of the benzyl group. Despite the facile inter­conversion of its different conformers (**A**–**D**), evidenced by a single resonance for the two pyrazole C—H protons in its ^1^H NMR spectrum (Fig. 4 ▸), **1** adopts a chiral crystal structure (in the achiral, yet non-centrosymmetric space group *P*2_1_; Flack, 2003 ▸). This must be the result of a more efficient crystal packing in the non-centrosymmetric space group (detailed in the next section) than in a centrosymmetric one.

## 3. Supra­molecular features

**Figure 5:**
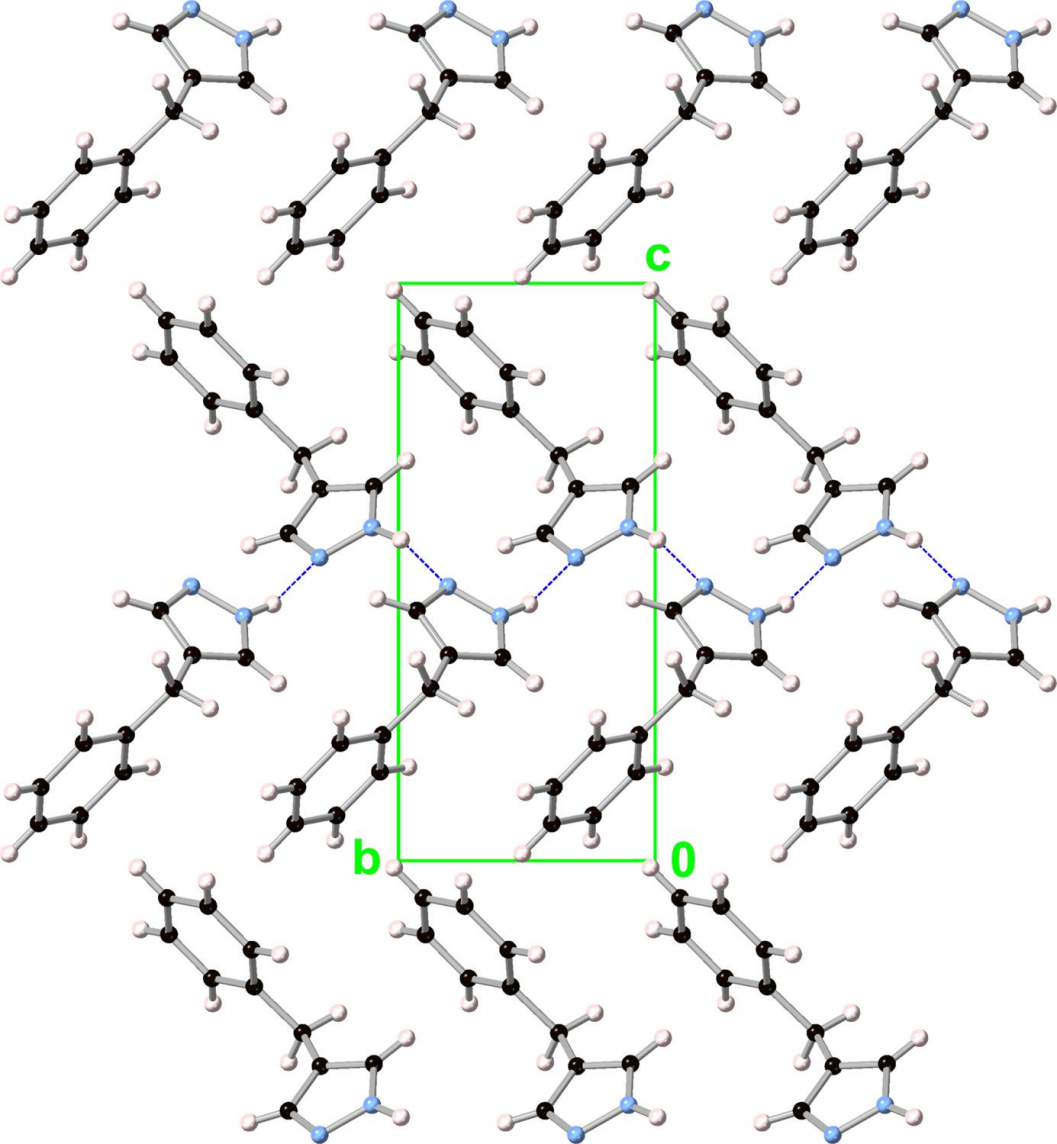
Packing diagram (viewed along the *a* axis) of 4-benzyl-1*H*-pyrazole (**1**).

The pz moieties in **1** are organized into layers along the *ab* plane, which consist of two symmetry-related (by a 2_1_ screw axis) halves (Fig. 5 ▸). Within each half, the pz moieties are all parallel to each other (crystallographically imposed) and are organized into parallel columns along both the *a* and *b* axes (which are orthogonal), with pz–pz inter­planar distances of 3.540 (4) and 2.184 (5) Å, and centroid–centroid distances of 5.6651 (5) and 5.7566 (6) Å, respectively. The two halves of the pz layer are connected by edge-to-face pz–pz inter­actions with dihedral angles of 44.59 (11)° and centroid–centroid distances of 4.3813 (12) Å (closest H⋯pz-plane and H⋯pz-centroid distances: 2.7021 (8) and 2.7052 (8) Å), as well as by N—H⋯N hydrogen bonding between pz moieties (Table 1 ▸), which leads to catemers along the *b* axis with pz/pz dihedral angles of 44.59 (11)° and centroid–centroid distances of 4.3813 (12) Å).

Similarly to the pz moieties, the phenyl moieties of **1** also form layers along the *ab* plane, with two 2_1_ screw axis-related halves comprised of parallel columns along both the *a* and *b* axes [Ph–Ph inter­planar distances of 2.557 (4) and 3.516 (4) Å, and centroid–centroid distances of 5.6651 (5) and 5.7566 (6) Å, respectively]. Edge-to-face Ph–Ph inter­actions connect the two halves of the Ph layer, with dihedral angles of 75.29 (9)° and centroid–centroid distances of 4.8833 (11) Å [closest H⋯Ph-plane and H⋯Ph-centroid distances: 2.7962 (10) and 2.8660 (7) Å].

The benzyl protons of **1** are involved in C—H⋯π hydrogen bonding with neighboring pz and Ph moieties, with H⋯pz/Ph-plane, H⋯pz/Ph-centroid and closest H⋯N/C distances of 2.6778 (16), 3.1016 (8), 2.7399 (17), and 2.503 (2), 3.3598 (9), 2.659 (2) Å, respectively.

**Figure 6:**
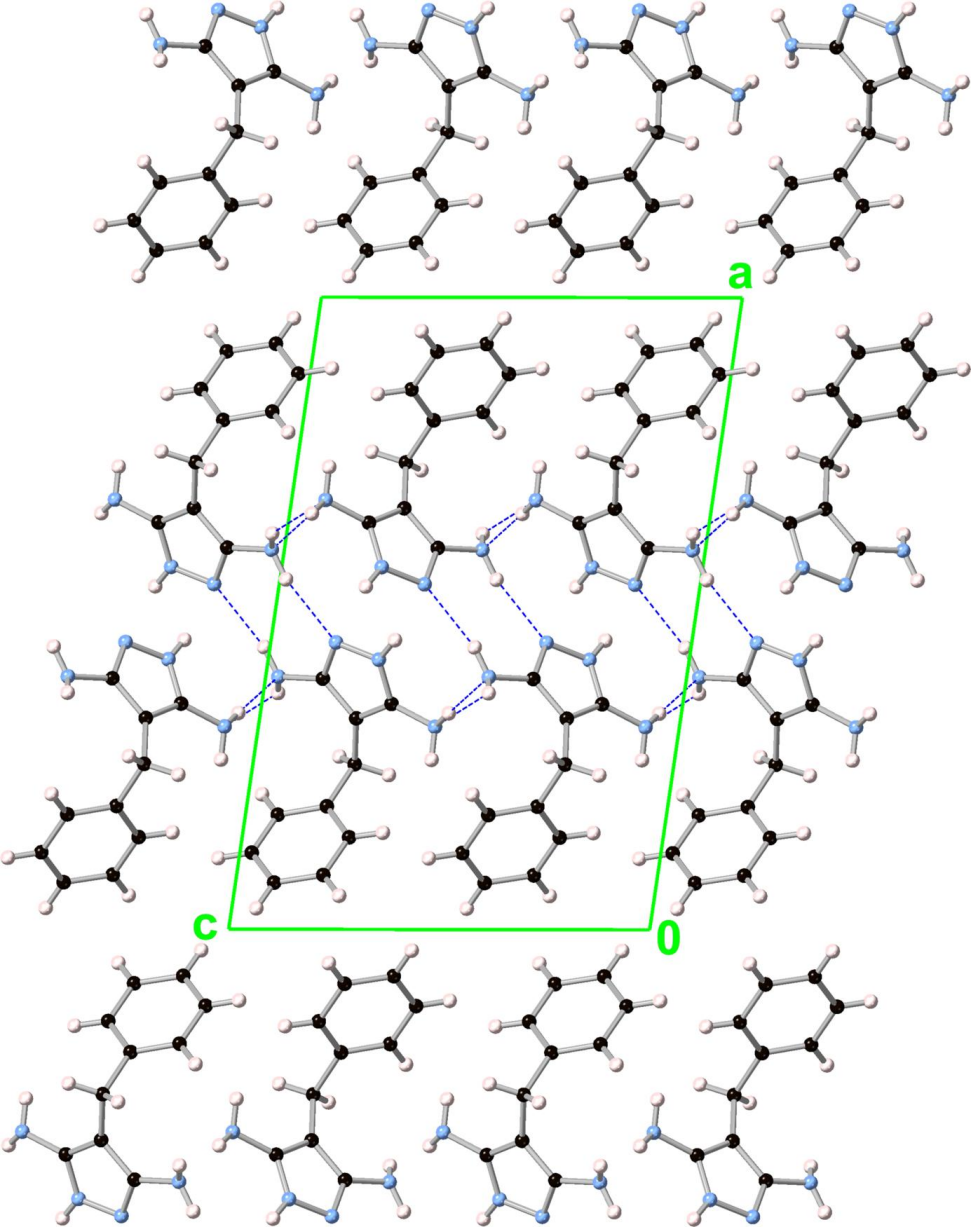
Packing diagram (viewed along the *b* axis) of 3,5-di­amino-4-benzyl-1*H*-pyrazole (**2**).

In **2**, the pz moieties form layers along the *bc* plane, which are comprised of two 2_1_ screw axis-related halves as in **1** (Fig. 6 ▸). Here, however, the pz moieties are only parallel within individual columns and in every second column, with dihedral angles between neighboring inter-columnar pz moieties of 85.71 (7)° [centroid–centroid distance: 5.9674 (8) Å]. Within each column, the pz–pz inter­planar and centroid–centroid distances are 3.4653 (18) and 4.7271 (7) Å, respectively. The two halves of the pz layer are connected by edge-to-face inter­actions characterized by dihedral angles of 85.71 (7)° and centroid–centroid distances of 4.5404 (9) Å [closest H⋯pz-plane, H⋯pz-centroid and H⋯N distances: 1.974 (16), 2.769 (16) and 2.108 (16) Å], as well as by N—H⋯N hydrogen bonding (Table 2 ▸). Unlike in **1**, this inter-layer hydrogen bonding in **2** occurs between NH_2_ donor and N(pz) acceptor moieties, while the N—H(pz) hydrogen atom is involved in an N—H⋯π inter­action. Within each half-layer, there are additional hydrogen bonds between neighboring NH_2_ groups, one on each side of the pz moieties (Table 2 ▸). Since there are five N—H hydrogen-bond donors but only four hydrogen-bond acceptors in **2**, one of the NH_2_ hydrogen atoms does not have a hydrogen-bond acceptor. Instead, an N—H⋯π inter­action is formed with a neighboring Ph moiety, with H⋯Ph-plane, H⋯Ph-centroid and closest H⋯C distances of 2.840 (15), 3.363 (15) and 2.956 (15) Å, respectively.

Similarly to **1**, the phenyl moieties of **2** form layers but along the *bc* plane, which are analogous to the layers formed by its pz moieties with dihedral angles of 85.07 (6)° between neighboring inter-columnar Ph moieties [centroid–centroid distance: 6.0946 (8) Å]. Within each column, the Ph–Ph inter­planar and centroid–centroid distances are 3.4833 (18) and 4.7271 (7) Å, respectively. Because the Ph moieties in neighboring columns are not parallel, two types of Ph–Ph inter­actions are present between the two halves of the Ph layer. Edge-to-face inter­actions are characterized by dihedral angles of 85.07 (6)° and centroid–centroid distances of 5.4925 (10) Å [closest H⋯Ph-plane and H⋯Ph-centroid distances: 2.8014 (3) and 3.4126 (6) Å], in addition to offset stacked inter­actions between parallel Ph moieties with inter-planar and centroid–centroid distances of 1.997 (3) and 6.0466 (13) Å, respectively.

In **2**, only one of the benzyl protons is involved in C—H⋯π hydrogen bonding with neighboring pz moieties, characterized by H⋯pz-plane, H⋯pz-centroid and closest H⋯C distances of 2.9081 (9), 3.2096 (5) and 2.9183 (11) Å, respectively.

## 4. Database survey

Two crystal structures of simple derivatives of 4-benzyl-1*H*-pyrazole are known, namely 3,5-dimethyl-4-benzyl-1*H*-pyrazole (**3**; Wang & Kong, 2011 ▸; CCDC refcode: OBUHOK) and 3,5-diphenyl-4-benzyl-1*H*-pyrazole (**4**; Huang *et al.*, 2007 ▸; CCDC refcode: XEYYAC). Inter­estingly, 3,5-dimethyl-4-benzyl-1*H*-pyrazole crystallizes in the same space group (*P*2_1_) as its parent compound, 4-benzyl-1*H*-pyrazole (**1**), instead of the centrosymmetric space group (*P*2_1_/*c*) of its structurally more similar 3,5-di­amino-4-benzyl-1*H*-pyrazole (**2**). This is likely due to the hydrogen-bond donor/acceptor ability of the NH_2_ groups of **2** compared to the CH_3_ groups of **3**.

Not only does **3** crystallize in the same space group as **1**, but it also adopts a very similar crystal packing in a unit cell of comparable dimensions [*a* = 6.2303 (6) Å; *b* = 5.5941 (5) Å; *c* = 15.1364 (15) Å; *β* = 97.049 (1)°]. Notably, its *c* axis is longer than in **1** [13.2321 (9) Å] to accommodate the bulkier CH_3_ group compared to H. The C—C—C bond angle of 66.3 (3)° between the pz, CH_2_ and Ph units is closer to that of **2** [65.65 (8)°], with a pz/Ph centroid–centroid distance of 4.6524 (2) Å, shorter than in both **1** and **2**. The dihedral angle of 78.65 (13)° between the pz and Ph units in **3** is in-between the values of **1** and **2**, with individual fold and twist angles of 60.60 (14) and 52.27 (16)°.

The supra­molecular features of **3** are similar to those of **1**, with a slightly expanded crystal packing due to the presence of the CH_3_ groups. Thus, the parallel columns along the *a* and *b* axes feature pz–pz inter­planar distances of 2.995 (8) and 3.514 (7) Å, and centroid–centroid distances of 6.2303 (6) and 5.5941 (5) Å, respectively. The edge-to-face orientation of the pz/pz pairs within the two halves of the pz layer is described by a dihedral angle of 77.83 (18)° and centroid–centroid distance of 5.8969 (19) Å, which are significantly larger than the corresponding values in **1** [44.59 (11)° and 4.3813 (12) Å]. The N—H⋯N hydrogen bonding between pz moieties leading to catemers along the *b* axis is characterized by N—H, H⋯N and N⋯N distances of 0.86, 2.09 and 2.946 (4) Å, with an N—H⋯N angle of 170°, pz/pz dihedral angle of 77.83 (18)° and centroid–centroid distance of 4.9789 (19) Å. The corresponding values for the Ph–Ph inter­actions along the *a* and *b* axes are 2.202 (11) and 3.772 (7) Å (inter­planar) and 6.2302 (6) and 5.5941 (5) Å (centroid–centroid), whereas between the two halves of the Ph layer the values are 84.8 (2)° (dihedral angle) and 5.066 (3) Å (centroid–centroid), with closest H⋯Ph-plane and H⋯Ph-centroid distances of 2.863 (3) and 3.1738 (17) Å. Similarly to **1**, the methyl and benzyl protons are involved in various C—H⋯π inter­actions with neighboring pz and Ph moieties.

3,5-Diphenyl-4-benzyl-1*H*-pyrazole (**4**) crystallizes in the centrosymmetric space group *P*2_1_/*c*. As opposed to **1**–**3**, however, the N—H⋯N hydrogen bonding between pz moieties does not lead to catemers. Instead, **4** forms hydrogen-bonded dimers, with an overall crystal packing very different from the ones of **1**–**3**.

## 5. Synthesis and crystallization

4-Benzyl-1*H*-pyrazole (**1**) was synthesized by reduction with hypo­phospho­rous acid of 3,5-di­amino-4-benzyl-1*H*-pyrazole (**2**) (Echevarría & Elguero, 1993 ▸), which in turn was obtained from benzyl­malono­nitrile by reaction with hydrazine hydrate (Vaquero *et al.*, 1987 ▸). The synthesis of benzyl­malono­nitrile by alkyl­ation of malono­nitrile with benzyl bromide provided the mono­benzyl­ated product contaminated with large amounts of di­benzyl­ated side product (Díez-Barra *et al.*, 1991 ▸). Therefore, an alternate method, by the reaction of malono­nitrile with benzaldehyde and reduction of the benzyl­idenemalono­nitrile inter­mediate with NaBH_4_ was used for the preparation of pure benzyl­malono­nitrile in high yield (Tayyari *et al.*, 2008 ▸). Single crystals were grown by recrystallization from hot *n*-heptane (**1**) or by vapor diffusion of benzene into a solution in pyridine at room temperature (**2**).

## 6. Refinement

Crystal data, data collection and structure refinement details are summarized in Table 3 ▸. C—H bond distances were constrained to 0.95 Å (pz and Ph) or 0.99 Å (CH_2_) and refined as riding. Positions of N-bound H atoms were freely refined. *U*_iso_(H) values were set to 1.2 or 1.5 times *U*_eq_(C/N) for H atoms.

## Supplementary Material

Crystal structure: contains datablock(s) 1, 2. DOI: 10.1107/S2056989024006182/vm2305sup1.cif

Structure factors: contains datablock(s) 1. DOI: 10.1107/S2056989024006182/vm23051sup2.hkl

Supporting information file. DOI: 10.1107/S2056989024006182/vm23051sup4.cdx

Structure factors: contains datablock(s) 2. DOI: 10.1107/S2056989024006182/vm23052sup3.hkl

Supporting information file. DOI: 10.1107/S2056989024006182/vm23052sup5.cdx

CCDC references: 2365130, 2365129

Additional supporting information:  crystallographic information; 3D view; checkCIF report

## Figures and Tables

**Figure 2:**
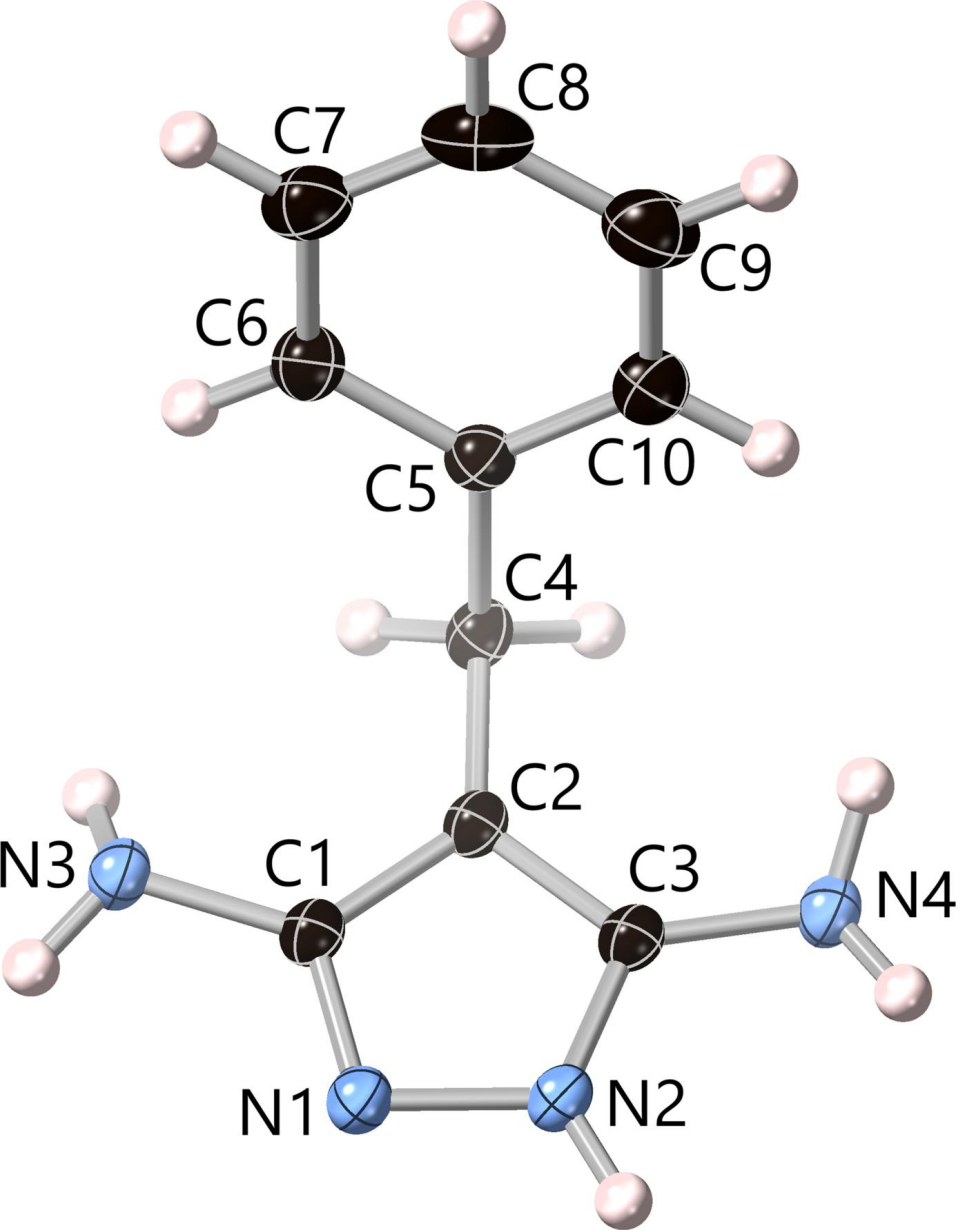
Displacement ellipsoid plot (50% probability) of the crystal structure of 3,5-di­amino-4-benzyl-1*H*-pyrazole (**2**), showing the contents of the asymmetric unit.

**Table 1:** Hydrogen-bond geometry (Å, °) for **1**[Chem scheme1]

*D*—H⋯*A*	*D*—H	H⋯*A*	*D*⋯*A*	*D*—H⋯*A*
N1—H1*N*⋯N2^i^	0.90 (2)	2.01 (2)	2.887 (2)	163 (2)

Symmetry code: (i) 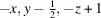.

**Table 2:** Hydrogen-bond geometry (Å, °) for **2**[Chem scheme1]

*D*—H⋯*A*	*D*—H	H⋯*A*	*D*⋯*A*	*D*—H⋯*A*
N2—H2⋯N1^i^	0.894 (16)	2.108 (16)	2.9912 (13)	169.7 (14)
N3—H3*A*⋯N1^ii^	0.898 (17)	2.240 (17)	3.1032 (14)	161.0 (13)
N3—H3*B*⋯N4^iii^	0.905 (17)	2.383 (16)	3.1521 (14)	142.8 (13)
N4—H4*A*⋯N3^iv^	0.905 (17)	2.140 (18)	3.0388 (14)	171.7 (14)

Symmetry codes: (i) 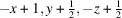; (ii) 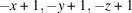; (iii) 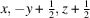; (iv) 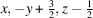.

**Table 3:** Experimental details

	**1**	**2**
Crystal data		
Chemical formula	C_10_H_10_N_2_	C_10_H_12_N_4_
*M*_r_	158.20	188.24
Crystal system, space group	Monoclinic, *P*2_1_	Monoclinic, *P*2_1_/*c*
Temperature (K)	150	150
*a*, *b*, *c* (Å)	5.6651 (5), 5.7566 (6), 13.2321 (9)	17.410 (2), 4.7271 (7), 11.4664 (15)
β (°)	101.732 (4)	98.247 (6)
*V* (Å^3^)	422.51 (6)	933.9 (2)
*Z*	2	4
Radiation type	Cu *K*α	Cu *K*α
μ (mm^−1^)	0.59	0.69
Crystal size (mm)	0.14 × 0.13 × 0.09	0.23 × 0.21 × 0.11

Data collection		
Diffractometer	Bruker AXS D8 Quest	Bruker AXS D8 Quest
Absorption correction	Multi-scan (*SADABS*; Krause *et al.*, 2015 ▸)	Multi-scan (*SADABS*; Krause *et al.*, 2015 ▸)
*T*_min_, *T*_max_	0.605, 0.754	0.543, 0.754
No. of measured, independent and observed [*I* > 2σ(*I*)] reflections	4021, 1612, 1549	10090, 1963, 1817
*R*_int_	0.052	0.063
(sin θ/λ)_max_ (Å^−1^)	0.639	0.638

Refinement		
*R*[*F*^2^ > 2σ(*F*^2^)], *wR*(*F*^2^), *S*	0.032, 0.084, 1.08	0.038, 0.095, 1.03
No. of reflections	1612	1963
No. of parameters	112	142
No. of restraints	1	0
H-atom treatment	H atoms treated by a mixture of independent and constrained refinement	H atoms treated by a mixture of independent and constrained refinement
Δρ_max_, Δρ_min_ (e Å^−3^)	0.15, −0.16	0.21, −0.20
Absolute structure	Flack *x* determined using 605 quotients [(*I*^+^)−(*I*^−^)]/[(*I*^+^)+(*I*^−^)] (Parsons *et al.*, 2013 ▸)	–
Absolute structure parameter	0.0 (3)	–

Computer programs: *APEX5* (Bruker, 2023 ▸), *SAINT* (Bruker, 2020 ▸), *SHELXT* (Sheldrick, 2015*a* ▸), *SHELXL2019/2* (Sheldrick, 2015*b* ▸), *ShelXle* (Hübschle *et al.*, 2011 ▸) and *CrystalMaker* (CrystalMaker Software, 2003 ▸).

## supplementary crystallographic information

### 4-Benzyl-1H-pyrazole (1) Crystal data

**Table:**

C_10_H_10_N_2_	*F*(000) = 168
*M**_r_* = 158.20	*D*_x_ = 1.244 Mg m^−^^3^
Monoclinic, *P*2_1_	Cu *K*α radiation, λ = 1.54178 Å
*a* = 5.6651 (5) Å	Cell parameters from 3320 reflections
*b* = 5.7566 (6) Å	θ = 3.4–78.8°
*c* = 13.2321 (9) Å	µ = 0.59 mm^−^^1^
β = 101.732 (4)°	*T* = 150 K
*V* = 422.51 (6) Å^3^	Plate, colourless
*Z* = 2	0.14 × 0.13 × 0.09 mm

### 4-Benzyl-1H-pyrazole (1) Data collection

**Table:**

Bruker AXS D8 Quest diffractometer	1612 independent reflections
Radiation source: I-mu-S 3.0 microsource X-ray tube	1549 reflections with *I* > 2σ(*I*)
HELIOS multilayer Montel optics monochromator	*R*_int_ = 0.052
Detector resolution: 7.4074 pixels mm^-1^	θ_max_ = 80.0°, θ_min_ = 3.4°
ω and phi scans	*h* = −6→5
Absorption correction: multi-scan (SADABS; Krause *et al.*, 2015)	*k* = −6→7
*T*_min_ = 0.605, *T*_max_ = 0.754	*l* = −16→16
4021 measured reflections	

### 4-Benzyl-1H-pyrazole (1) Refinement

**Table:**

Refinement on *F*^2^	Secondary atom site location: difference Fourier map
Least-squares matrix: full	Hydrogen site location: mixed
*R*[*F*^2^ > 2σ(*F*^2^)] = 0.032	H atoms treated by a mixture of independent and constrained refinement
*wR*(*F*^2^) = 0.084	*w* = 1/[σ^2^(*F*_o_^2^) + (0.0261*P*)^2^ + 0.0328*P*] where *P* = (*F*_o_^2^ + 2*F*_c_^2^)/3
*S* = 1.08	(Δ/σ)_max_ < 0.001
1612 reflections	Δρ_max_ = 0.14 e Å^−^^3^
112 parameters	Δρ_min_ = −0.16 e Å^−^^3^
1 restraint	Absolute structure: Flack *x* determined using 605 quotients [(*I*^+^)-(*I*^-^)]/[(*I*^+^)+(*I*^-^)] (Parsons *et al.*, 2013)
Primary atom site location: dual	Absolute structure parameter: 0.0 (3)

### 4-Benzyl-1H-pyrazole (1) Special details

**Table:**

Geometry. All esds (except the esd in the dihedral angle between two l.s. planes) are estimated using the full covariance matrix. The cell esds are taken into account individually in the estimation of esds in distances, angles and torsion angles; correlations between esds in cell parameters are only used when they are defined by crystal symmetry. An approximate (isotropic) treatment of cell esds is used for estimating esds involving l.s. planes.

### 4-Benzyl-1H-pyrazole (1) Fractional atomic coordinates and isotropic or equivalent isotropic displacement parameters (Å^2^)

**Table:**

	*x*	*y*	*z*	*U*_iso_*/*U*_eq_	
N1	0.2067 (3)	0.1035 (3)	0.57682 (11)	0.0298 (3)	
H1N	0.097 (4)	−0.009 (4)	0.5558 (18)	0.045*	
N2	0.1810 (3)	0.3078 (3)	0.52511 (10)	0.0304 (3)	
C1	0.4146 (3)	0.0962 (3)	0.64801 (12)	0.0296 (4)	
H1	0.469928	−0.030094	0.692813	0.036*	
C2	0.5319 (3)	0.3046 (3)	0.64400 (12)	0.0281 (4)	
C3	0.3770 (3)	0.4290 (3)	0.56670 (13)	0.0294 (4)	
H3	0.408236	0.582314	0.546294	0.035*	
C4	0.7775 (3)	0.3749 (3)	0.70179 (14)	0.0333 (4)	
H4A	0.872243	0.428501	0.651089	0.040*	
H4B	0.858928	0.235376	0.736236	0.040*	
C5	0.7846 (3)	0.5626 (3)	0.78212 (12)	0.0285 (4)	
C6	0.9684 (3)	0.7272 (3)	0.79620 (14)	0.0342 (4)	
H6	1.083272	0.724351	0.752955	0.041*	
C7	0.9862 (4)	0.8950 (4)	0.87229 (15)	0.0413 (5)	
H7	1.113464	1.005361	0.881061	0.050*	
C8	0.8206 (4)	0.9031 (4)	0.93542 (15)	0.0433 (5)	
H8	0.833562	1.017860	0.987870	0.052*	
C9	0.6349 (3)	0.7423 (4)	0.92160 (14)	0.0389 (5)	
H9	0.518731	0.748123	0.964163	0.047*	
C10	0.6177 (3)	0.5727 (3)	0.84595 (13)	0.0329 (4)	
H10	0.490632	0.462217	0.837655	0.039*	

### 4-Benzyl-1H-pyrazole (1) Atomic displacement parameters (Å^2^)

**Table:**

	*U*^11^	*U*^22^	*U*^33^	*U*^12^	*U*^13^	*U*^23^
N1	0.0313 (7)	0.0251 (7)	0.0323 (7)	−0.0007 (6)	0.0050 (6)	−0.0005 (6)
N2	0.0325 (7)	0.0279 (7)	0.0303 (7)	0.0030 (6)	0.0052 (6)	0.0012 (6)
C1	0.0346 (9)	0.0248 (8)	0.0282 (7)	0.0022 (7)	0.0034 (6)	0.0014 (6)
C2	0.0298 (8)	0.0280 (8)	0.0270 (7)	0.0015 (7)	0.0069 (6)	−0.0028 (7)
C3	0.0330 (9)	0.0236 (8)	0.0321 (8)	0.0014 (7)	0.0082 (6)	0.0003 (6)
C4	0.0287 (9)	0.0344 (10)	0.0360 (8)	0.0025 (7)	0.0045 (7)	−0.0039 (7)
C5	0.0263 (8)	0.0299 (9)	0.0269 (7)	0.0013 (7)	−0.0004 (6)	0.0035 (6)
C6	0.0290 (9)	0.0365 (9)	0.0358 (9)	−0.0019 (8)	0.0033 (7)	0.0060 (7)
C7	0.0398 (11)	0.0341 (9)	0.0443 (10)	−0.0084 (8)	−0.0047 (8)	0.0011 (8)
C8	0.0461 (12)	0.0386 (10)	0.0394 (10)	0.0040 (9)	−0.0052 (8)	−0.0093 (8)
C9	0.0370 (10)	0.0457 (12)	0.0337 (9)	0.0048 (9)	0.0062 (7)	−0.0040 (8)
C10	0.0294 (8)	0.0348 (9)	0.0332 (8)	−0.0019 (7)	0.0033 (6)	0.0008 (7)

### 4-Benzyl-1H-pyrazole (1) Geometric parameters (Å, º)

**Table:**

N1—C1	1.351 (2)	C5—C10	1.391 (3)
N1—N2	1.353 (2)	C5—C6	1.392 (2)
N1—H1N	0.90 (2)	C6—C7	1.384 (3)
N2—C3	1.332 (2)	C6—H6	0.9500
C1—C2	1.378 (3)	C7—C8	1.378 (3)
C1—H1	0.9500	C7—H7	0.9500
C2—C3	1.401 (2)	C8—C9	1.385 (3)
C2—C4	1.500 (2)	C8—H8	0.9500
C3—H3	0.9500	C9—C10	1.388 (3)
C4—C5	1.511 (2)	C9—H9	0.9500
C4—H4A	0.9900	C10—H10	0.9500
C4—H4B	0.9900		
			
C1—N1—N2	111.63 (14)	C10—C5—C6	118.22 (16)
C1—N1—H1N	130.0 (15)	C10—C5—C4	122.19 (15)
N2—N1—H1N	118.0 (15)	C6—C5—C4	119.55 (16)
C3—N2—N1	104.56 (13)	C7—C6—C5	120.94 (18)
N1—C1—C2	107.75 (15)	C7—C6—H6	119.5
N1—C1—H1	126.1	C5—C6—H6	119.5
C2—C1—H1	126.1	C8—C7—C6	120.44 (19)
C1—C2—C3	103.75 (15)	C8—C7—H7	119.8
C1—C2—C4	128.29 (16)	C6—C7—H7	119.8
C3—C2—C4	127.76 (17)	C7—C8—C9	119.31 (18)
N2—C3—C2	112.30 (15)	C7—C8—H8	120.3
N2—C3—H3	123.8	C9—C8—H8	120.3
C2—C3—H3	123.8	C8—C9—C10	120.38 (18)
C2—C4—C5	116.15 (15)	C8—C9—H9	119.8
C2—C4—H4A	108.2	C10—C9—H9	119.8
C5—C4—H4A	108.2	C9—C10—C5	120.69 (17)
C2—C4—H4B	108.2	C9—C10—H10	119.7
C5—C4—H4B	108.2	C5—C10—H10	119.7
H4A—C4—H4B	107.4		
			
C1—N1—N2—C3	−0.69 (18)	C2—C4—C5—C6	−143.18 (17)
N2—N1—C1—C2	0.29 (19)	C10—C5—C6—C7	0.6 (3)
N1—C1—C2—C3	0.21 (18)	C4—C5—C6—C7	−177.05 (16)
N1—C1—C2—C4	−174.91 (16)	C5—C6—C7—C8	−0.4 (3)
N1—N2—C3—C2	0.84 (18)	C6—C7—C8—C9	−0.4 (3)
C1—C2—C3—N2	−0.67 (19)	C7—C8—C9—C10	0.9 (3)
C4—C2—C3—N2	174.49 (15)	C8—C9—C10—C5	−0.7 (3)
C1—C2—C4—C5	−112.26 (19)	C6—C5—C10—C9	−0.1 (3)
C3—C2—C4—C5	73.7 (2)	C4—C5—C10—C9	177.52 (16)
C2—C4—C5—C10	39.2 (2)		

### 4-Benzyl-1H-pyrazole (1) Hydrogen-bond geometry (Å, º)

**Table:**

*D*—H···*A*	*D*—H	H···*A*	*D*···*A*	*D*—H···*A*
N1—H1*N*···N2^i^	0.90 (2)	2.01 (2)	2.887 (2)	163 (2)

Symmetry code: (i) −*x*, *y*−1/2, −*z*+1.

### 3,5-Diamino-4-benzyl-1H-pyrazole (2) Crystal data

**Table:**

C_10_H_12_N_4_	*F*(000) = 400
*M**_r_* = 188.24	*D*_x_ = 1.339 Mg m^−^^3^
Monoclinic, *P*2_1_/*c*	Cu *K*α radiation, λ = 1.54178 Å
*a* = 17.410 (2) Å	Cell parameters from 7332 reflections
*b* = 4.7271 (7) Å	θ = 5.1–79.3°
*c* = 11.4664 (15) Å	µ = 0.69 mm^−^^1^
β = 98.247 (6)°	*T* = 150 K
*V* = 933.9 (2) Å^3^	Block, colourless
*Z* = 4	0.23 × 0.21 × 0.11 mm

### 3,5-Diamino-4-benzyl-1H-pyrazole (2) Data collection

**Table:**

Bruker AXS D8 Quest diffractometer	1963 independent reflections
Radiation source: I-mu-S 3.0 microsource X-ray tube	1817 reflections with *I* > 2σ(*I*)
HELIOS multilayer Montel optics monochromator	*R*_int_ = 0.063
Detector resolution: 7.4074 pixels mm^-1^	θ_max_ = 79.8°, θ_min_ = 5.1°
ω and phi scans	*h* = −22→21
Absorption correction: multi-scan (SADABS; Krause *et al.*, 2015)	*k* = −5→5
*T*_min_ = 0.543, *T*_max_ = 0.754	*l* = −14→14
10090 measured reflections	

### 3,5-Diamino-4-benzyl-1H-pyrazole (2) Refinement

**Table:**

Refinement on *F*^2^	Primary atom site location: dual
Least-squares matrix: full	Secondary atom site location: difference Fourier map
*R*[*F*^2^ > 2σ(*F*^2^)] = 0.038	Hydrogen site location: mixed
*wR*(*F*^2^) = 0.095	H atoms treated by a mixture of independent and constrained refinement
*S* = 1.03	*w* = 1/[σ^2^(*F*_o_^2^) + (0.0335*P*)^2^ + 0.2938*P*] where *P* = (*F*_o_^2^ + 2*F*_c_^2^)/3
1963 reflections	(Δ/σ)_max_ < 0.001
142 parameters	Δρ_max_ = 0.21 e Å^−^^3^
0 restraints	Δρ_min_ = −0.20 e Å^−^^3^

### 3,5-Diamino-4-benzyl-1H-pyrazole (2) Special details

**Table:**

Geometry. All esds (except the esd in the dihedral angle between two l.s. planes) are estimated using the full covariance matrix. The cell esds are taken into account individually in the estimation of esds in distances, angles and torsion angles; correlations between esds in cell parameters are only used when they are defined by crystal symmetry. An approximate (isotropic) treatment of cell esds is used for estimating esds involving l.s. planes.

### 3,5-Diamino-4-benzyl-1H-pyrazole (2) Fractional atomic coordinates and isotropic or equivalent isotropic displacement parameters (Å^2^)

**Table:**

	*x*	*y*	*z*	*U*_iso_*/*U*_eq_	
N1	0.45388 (5)	0.6148 (2)	0.34503 (8)	0.0242 (2)	
N2	0.42688 (6)	0.7374 (2)	0.23732 (8)	0.0244 (2)	
H2	0.4576 (9)	0.859 (3)	0.2061 (14)	0.037*	
N3	0.39960 (6)	0.2960 (2)	0.47002 (8)	0.0245 (2)	
H3A	0.4465 (10)	0.288 (3)	0.5142 (14)	0.037*	
H3B	0.3739 (9)	0.129 (4)	0.4651 (14)	0.037*	
N4	0.32068 (6)	0.7197 (2)	0.08053 (8)	0.0253 (2)	
H4A	0.3421 (9)	0.876 (4)	0.0527 (14)	0.038*	
H4B	0.2685 (10)	0.736 (3)	0.0774 (14)	0.038*	
C1	0.39646 (6)	0.4405 (2)	0.36362 (9)	0.0214 (2)	
C2	0.33394 (6)	0.4435 (2)	0.27059 (9)	0.0215 (2)	
C3	0.35673 (6)	0.6383 (2)	0.19157 (9)	0.0218 (2)	
C4	0.26130 (6)	0.2683 (2)	0.25826 (9)	0.0240 (2)	
H4C	0.273491	0.083850	0.297498	0.029*	
H4D	0.244323	0.230743	0.173545	0.029*	
C5	0.19460 (6)	0.4035 (2)	0.30979 (9)	0.0241 (2)	
C6	0.18121 (7)	0.3371 (3)	0.42321 (10)	0.0302 (3)	
H6	0.213624	0.203060	0.468164	0.036*	
C7	0.12111 (7)	0.4639 (3)	0.47196 (11)	0.0364 (3)	
H7	0.112630	0.415162	0.549475	0.044*	
C8	0.07384 (8)	0.6599 (3)	0.40819 (14)	0.0407 (3)	
H8	0.033299	0.748953	0.441860	0.049*	
C9	0.08595 (8)	0.7262 (3)	0.29431 (15)	0.0450 (4)	
H9	0.053343	0.859940	0.249479	0.054*	
C10	0.14562 (7)	0.5973 (3)	0.24591 (12)	0.0359 (3)	
H10	0.153077	0.642532	0.167597	0.043*	

### 3,5-Diamino-4-benzyl-1H-pyrazole (2) Atomic displacement parameters (Å^2^)

**Table:**

	*U*^11^	*U*^22^	*U*^33^	*U*^12^	*U*^13^	*U*^23^
N1	0.0249 (5)	0.0267 (5)	0.0216 (4)	0.0011 (4)	0.0054 (4)	0.0021 (3)
N2	0.0252 (5)	0.0264 (5)	0.0225 (4)	−0.0012 (4)	0.0070 (4)	0.0042 (3)
N3	0.0280 (5)	0.0265 (5)	0.0192 (4)	−0.0016 (4)	0.0036 (4)	0.0026 (4)
N4	0.0292 (5)	0.0270 (5)	0.0203 (4)	0.0014 (4)	0.0057 (4)	0.0028 (3)
C1	0.0251 (5)	0.0216 (5)	0.0186 (5)	0.0023 (4)	0.0066 (4)	−0.0020 (4)
C2	0.0247 (5)	0.0220 (5)	0.0187 (5)	0.0010 (4)	0.0061 (4)	−0.0012 (4)
C3	0.0252 (5)	0.0220 (5)	0.0194 (5)	0.0023 (4)	0.0074 (4)	−0.0017 (4)
C4	0.0287 (6)	0.0245 (5)	0.0191 (5)	−0.0031 (4)	0.0044 (4)	−0.0018 (4)
C5	0.0232 (5)	0.0254 (5)	0.0233 (5)	−0.0058 (4)	0.0022 (4)	−0.0024 (4)
C6	0.0301 (6)	0.0383 (6)	0.0222 (5)	−0.0012 (5)	0.0032 (4)	−0.0021 (5)
C7	0.0321 (6)	0.0488 (7)	0.0296 (6)	−0.0066 (5)	0.0090 (5)	−0.0091 (5)
C8	0.0269 (6)	0.0408 (7)	0.0567 (8)	−0.0031 (5)	0.0144 (6)	−0.0124 (6)
C9	0.0302 (7)	0.0403 (7)	0.0649 (10)	0.0055 (5)	0.0080 (6)	0.0119 (7)
C10	0.0306 (6)	0.0392 (7)	0.0381 (7)	0.0000 (5)	0.0062 (5)	0.0115 (5)

### 3,5-Diamino-4-benzyl-1H-pyrazole (2) Geometric parameters (Å, º)

**Table:**

N1—C1	1.3360 (14)	C4—H4C	0.9900
N1—N2	1.3839 (13)	C4—H4D	0.9900
N2—C3	1.3425 (15)	C5—C10	1.3869 (17)
N2—H2	0.894 (16)	C5—C6	1.3896 (15)
N3—C1	1.3924 (13)	C6—C7	1.3906 (17)
N3—H3A	0.898 (17)	C6—H6	0.9500
N3—H3B	0.905 (17)	C7—C8	1.378 (2)
N4—C3	1.3906 (14)	C7—H7	0.9500
N4—H4A	0.905 (17)	C8—C9	1.388 (2)
N4—H4B	0.907 (17)	C8—H8	0.9500
C1—C2	1.4106 (15)	C9—C10	1.3871 (19)
C2—C3	1.3892 (14)	C9—H9	0.9500
C2—C4	1.5012 (15)	C10—H10	0.9500
C4—C5	1.5174 (15)		
			
C1—N1—N2	103.56 (9)	C2—C4—H4D	108.7
C3—N2—N1	112.10 (9)	C5—C4—H4D	108.7
C3—N2—H2	129.1 (10)	H4C—C4—H4D	107.6
N1—N2—H2	118.7 (10)	C10—C5—C6	118.05 (11)
C1—N3—H3A	115.6 (10)	C10—C5—C4	121.30 (10)
C1—N3—H3B	114.6 (10)	C6—C5—C4	120.66 (10)
H3A—N3—H3B	113.7 (14)	C5—C6—C7	121.06 (12)
C3—N4—H4A	113.2 (10)	C5—C6—H6	119.5
C3—N4—H4B	112.2 (10)	C7—C6—H6	119.5
H4A—N4—H4B	112.1 (14)	C8—C7—C6	120.20 (12)
N1—C1—N3	120.41 (10)	C8—C7—H7	119.9
N1—C1—C2	112.78 (9)	C6—C7—H7	119.9
N3—C1—C2	126.62 (10)	C7—C8—C9	119.43 (12)
C3—C2—C1	103.77 (9)	C7—C8—H8	120.3
C3—C2—C4	128.31 (10)	C9—C8—H8	120.3
C1—C2—C4	127.88 (10)	C10—C9—C8	120.05 (13)
N2—C3—C2	107.78 (9)	C10—C9—H9	120.0
N2—C3—N4	121.81 (10)	C8—C9—H9	120.0
C2—C3—N4	130.32 (10)	C5—C10—C9	121.20 (12)
C2—C4—C5	114.35 (9)	C5—C10—H10	119.4
C2—C4—H4C	108.7	C9—C10—H10	119.4
C5—C4—H4C	108.7		
			
C1—N1—N2—C3	−1.02 (11)	C3—C2—C4—C5	−92.03 (13)
N2—N1—C1—N3	−174.26 (9)	C1—C2—C4—C5	90.56 (13)
N2—N1—C1—C2	0.95 (11)	C2—C4—C5—C10	83.88 (13)
N1—C1—C2—C3	−0.55 (12)	C2—C4—C5—C6	−95.73 (12)
N3—C1—C2—C3	174.30 (10)	C10—C5—C6—C7	−0.83 (18)
N1—C1—C2—C4	177.35 (10)	C4—C5—C6—C7	178.80 (11)
N3—C1—C2—C4	−7.80 (17)	C5—C6—C7—C8	−0.36 (19)
N1—N2—C3—C2	0.72 (12)	C6—C7—C8—C9	1.0 (2)
N1—N2—C3—N4	−176.05 (9)	C7—C8—C9—C10	−0.5 (2)
C1—C2—C3—N2	−0.11 (11)	C6—C5—C10—C9	1.34 (19)
C4—C2—C3—N2	−178.00 (10)	C4—C5—C10—C9	−178.28 (12)
C1—C2—C3—N4	176.29 (10)	C8—C9—C10—C5	−0.7 (2)
C4—C2—C3—N4	−1.60 (18)		

### 3,5-Diamino-4-benzyl-1H-pyrazole (2) Hydrogen-bond geometry (Å, º)

**Table:**

*D*—H···*A*	*D*—H	H···*A*	*D*···*A*	*D*—H···*A*
N2—H2···N1^i^	0.894 (16)	2.108 (16)	2.9912 (13)	169.7 (14)
N3—H3*A*···N1^ii^	0.898 (17)	2.240 (17)	3.1032 (14)	161.0 (13)
N3—H3*B*···N4^iii^	0.905 (17)	2.383 (16)	3.1521 (14)	142.8 (13)
N4—H4*A*···N3^iv^	0.905 (17)	2.140 (18)	3.0388 (14)	171.7 (14)

Symmetry codes: (i) −*x*+1, *y*+1/2, −*z*+1/2; (ii) −*x*+1, −*y*+1, −*z*+1; (iii) *x*, −*y*+1/2, *z*+1/2; (iv) *x*, −*y*+3/2, *z*−1/2.
